# Supplementary material for: Blood Vessel Patterning on Retinal Astrocytes Requires Endothelial Flt-1 (VEGFR-1)
Source: J Dev Biol. 2019 Sep 7;7(3):18. doi: 10.3390/jdb7030018 (PMC6787756; doi:10.3390/jdb7030018)
Supplement: Supplementary file 1 [file jdb-07-00018-s001.zip › ChappellBautch_JDB_SuppFILES-FINAL/ChappellBautch_JDB_SuppFIGURES_FINAL.pdf]

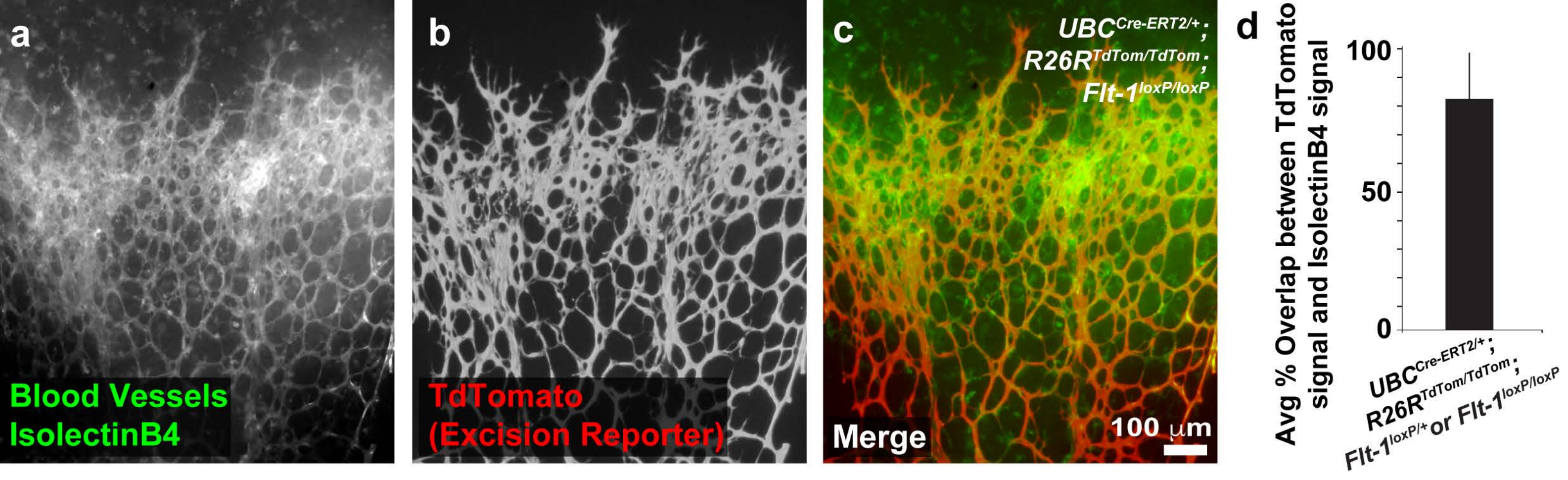
a
Blood Vessels
IsolectinB4
b
TdTomato
(Excision Reporter)
c
UBC^Cre-ERT2/+;
R26R^TdTom/TdTom;
Flt-1^loxP/loxP
Merge
100 μm
d
Avg % Overlap between TdTomato signal and IsolectinB4 signal
100
50
0
UBC^Cre-ERT2/+;
R26R^TdTom/TdTom;
Flt-1^loxP/+ or Flt-1^loxP/loxP

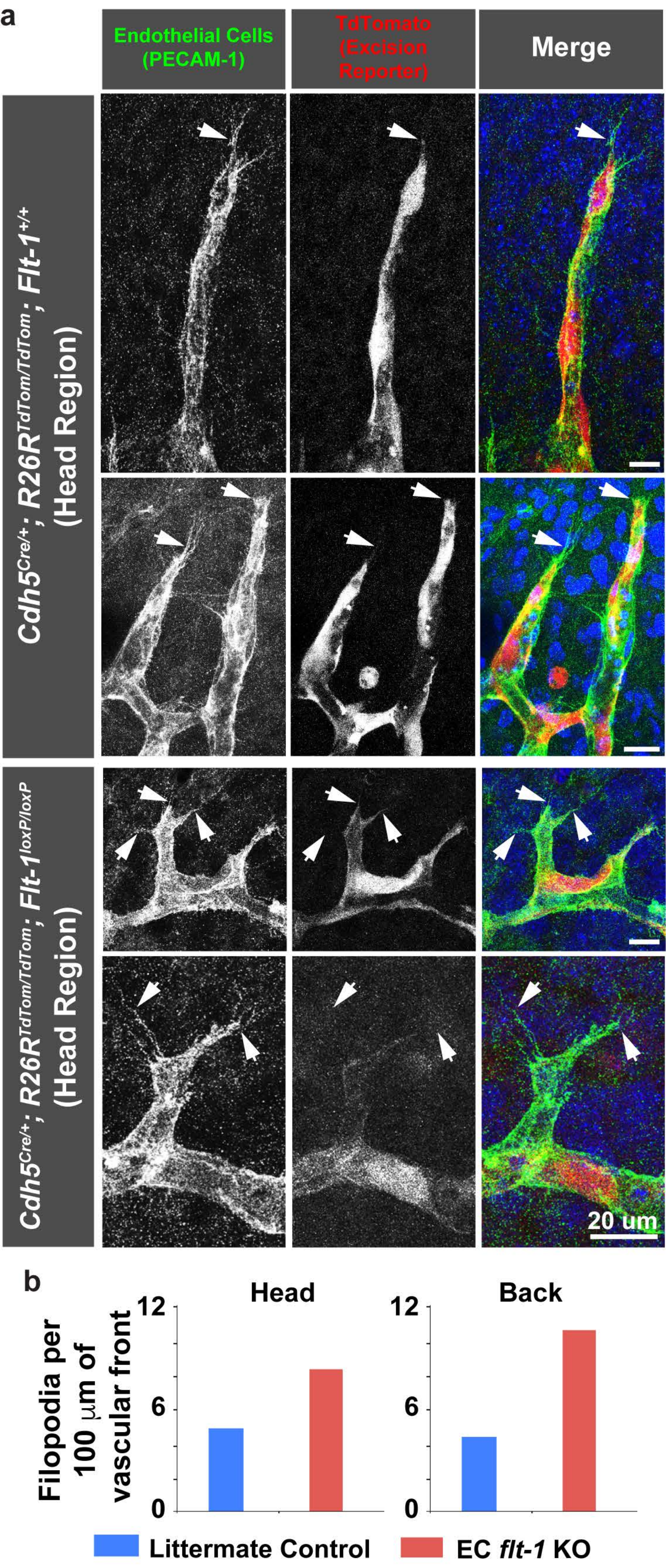

a
Endothelial Cells (PECAM-1)
TdTomato (Excision Reporter)
Merge
Cdh5Cre/+; R26RTdTom/TdTom; Flt-1+/+ (Head Region)
Cdh5Cre/+; R26RTdTom/TdTom; Flt-1loxP/loxP (Head Region)
20 um
b
Head
Back
Filopodia per 100 μm of vascular front
12
6
0
Littermate Control
EC flt-1 KO

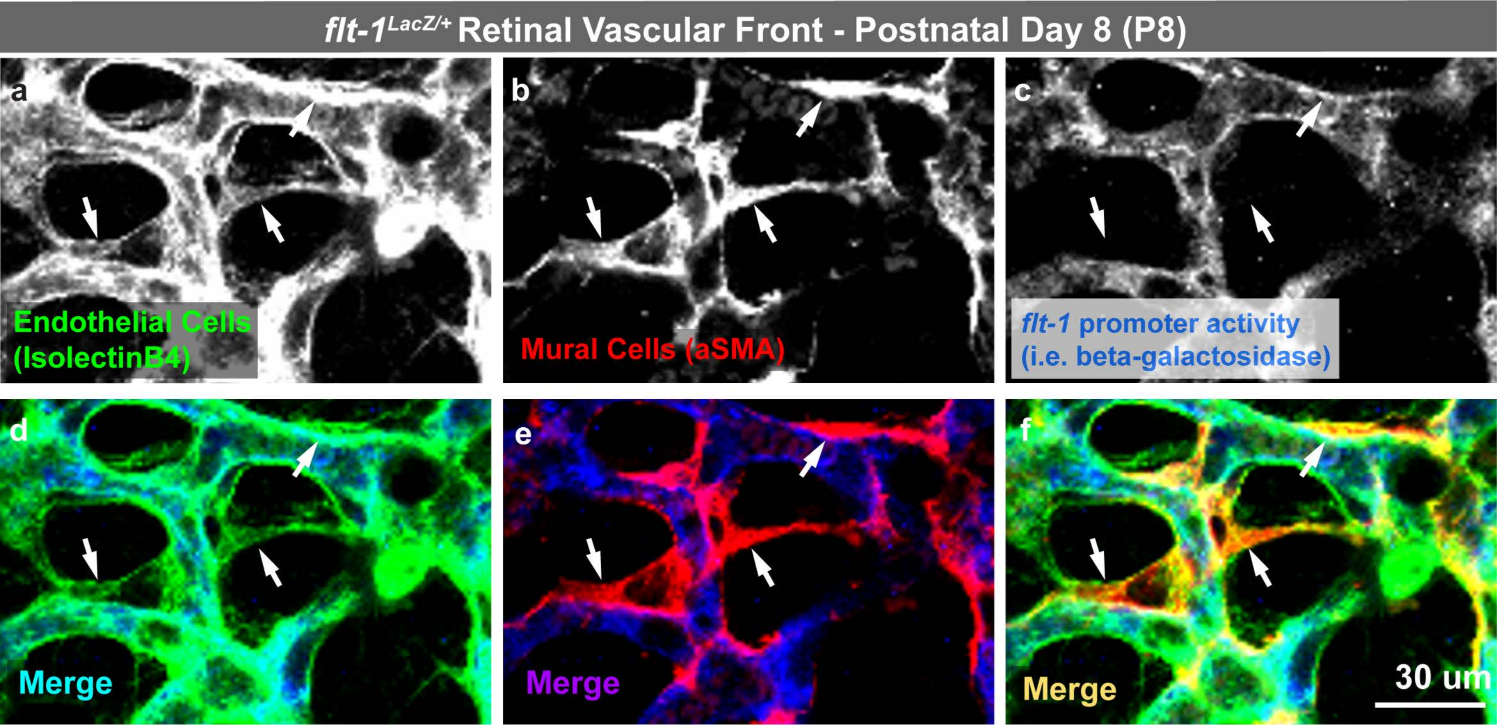
flt-1 LacZ/+ Retinal Vascular Front - Postnatal Day 8 (P8)
a
b
c
Endothelial Cells
(IsolectinB4)
Mural Cells (aSMA)
flt-1 promoter activity
(i.e. beta-galactosidase)
d
e
f
Merge
Merge
Merge
30 um

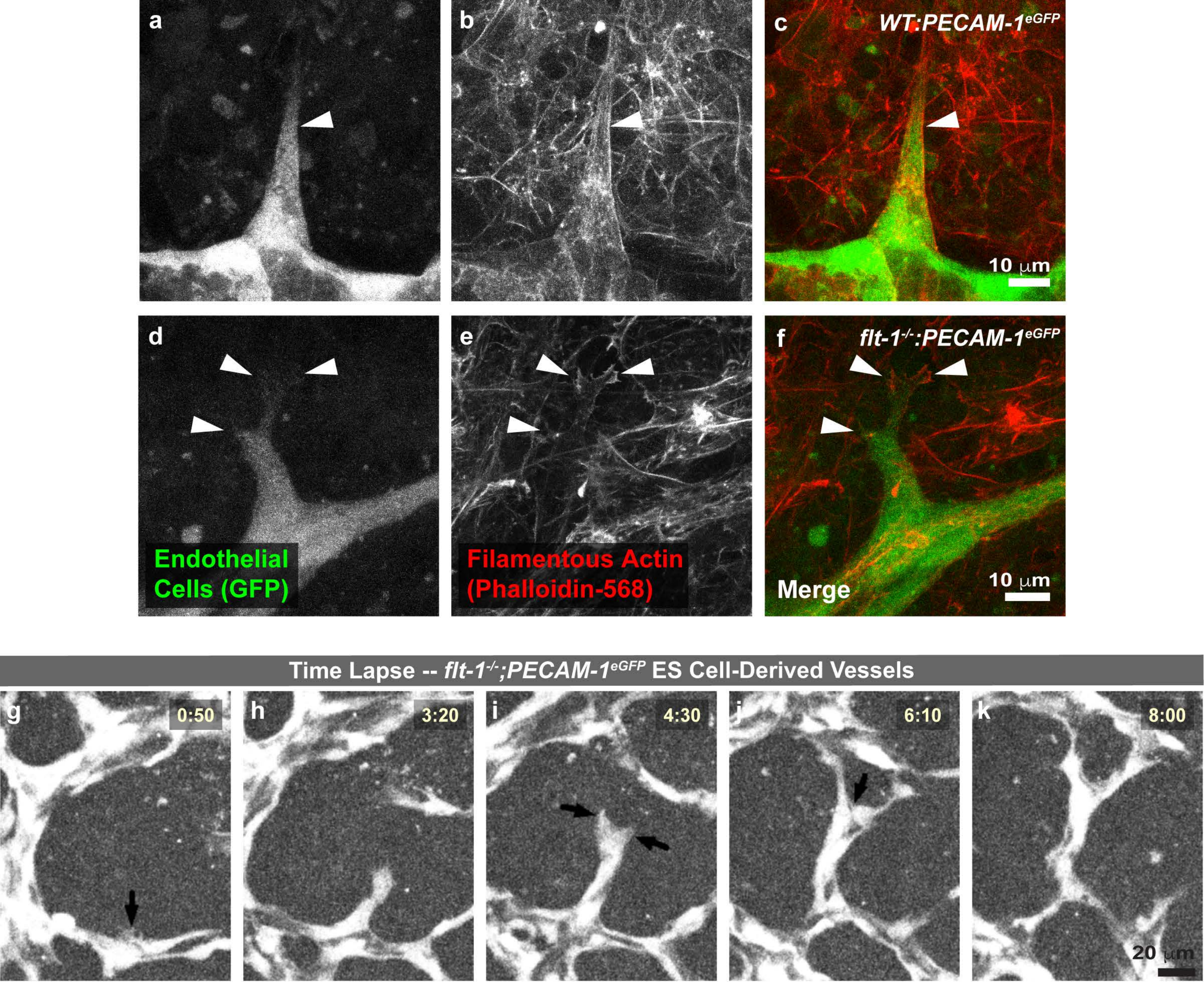
a
b
c
WT:PECAM-1eGFP
10 μm
d
e
f
flt-1-/-:PECAM-1eGFP
Endothelial Cells (GFP)
Filamentous Actin (Phalloidin-568)
Merge
10 μm
Time Lapse -- flt-1-/-;PECAM-1eGFP ES Cell-Derived Vessels
g
0:50
h
3:20
i
4:30
j
6:10
k
8:00
20 μm

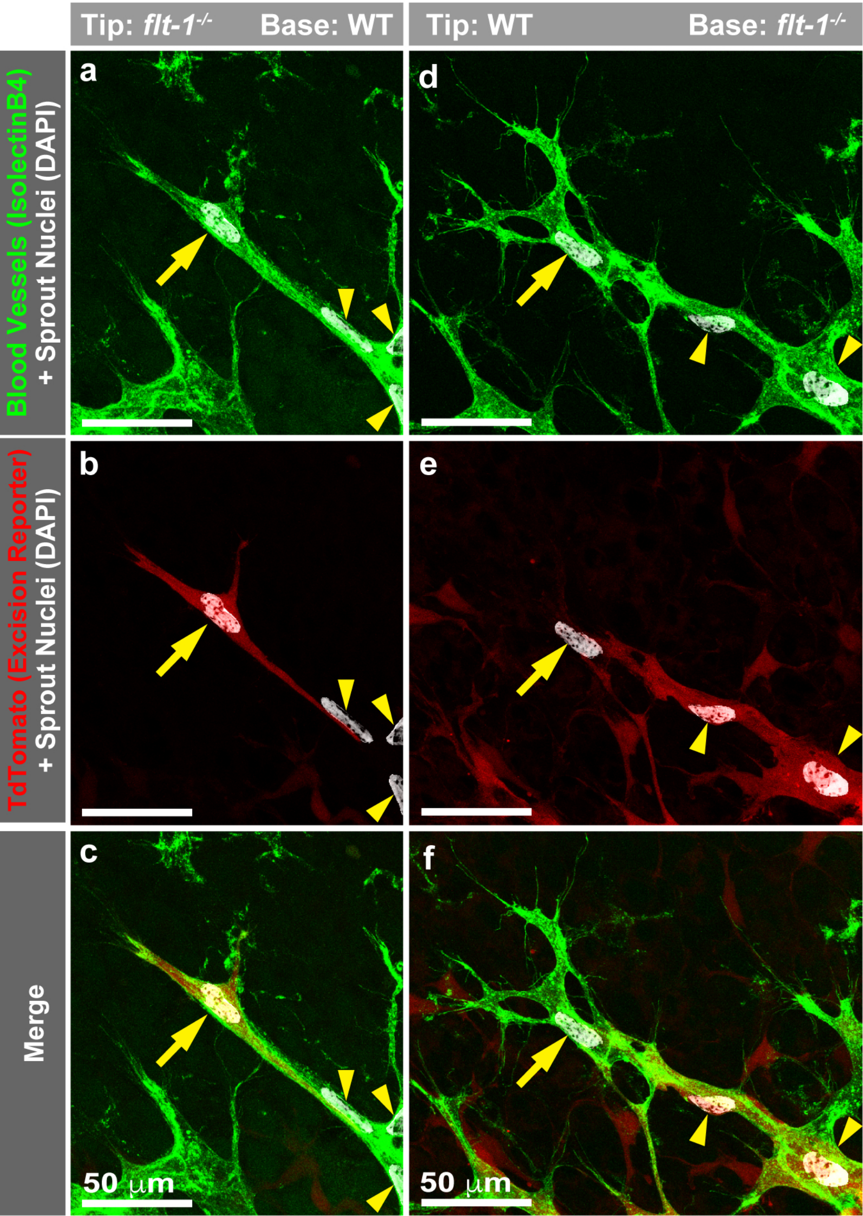

Tip: flt-1-/-
Base: WT
Tip: WT
Base: flt-1-/-
Blood Vessels (IsolectinB4) + Sprout Nuclei (DAPI)
TdTomato (Excision Reporter) + Sprout Nuclei (DAPI)
Merge
a
b
c
d
e
f
50 μm
50 μm
