# Supplementary material for: Blood Vessel Patterning on Retinal Astrocytes Requires Endothelial Flt-1 (VEGFR-1)
Source: J Dev Biol. 2019 Sep 7;7(3):18. doi: 10.3390/jdb7030018 (PMC6787756; doi:10.3390/jdb7030018)
Supplement: Supplementary file 1 [file jdb-07-00018-s001.zip › ChappellBautch_JDB_SuppFILES-FINAL/ChappellBautch_JDB_SuppINFO_FINAL.pdf]

Flt-1 Guides Vessel Patterning on Retinal Astrocytes

Online Resource

MATERIALS AND METHODS

Mouse Husbandry

As described previously [1], mice (*Mus musculus*) with the *Tg(UBC$^{Cre\text{-}ERT2}$)* gene (Jackson Laboratory #007001) or the *Cdh5-Cre* gene (Jackson Laboratory #006137) were bred with 2 additional mouse lines: 1) one line harboring a reporter gene for Cre recombinase (Cre)-mediated recombination, specifically *R26R$^{TdTom/TdTom}$* [*Gt(ROSA)26Sor$^{tm14(CAG\text{-}tdTomato)Hze}$*, Jackson Laboratory #007914], and 2) a line with *loxP* sites flanking the first exon of the *flt-1* gene [*Flt-1$^{loxP/loxP}$* (from Napoleone Ferrara, University of California, San Diego; formerly at Genentech)] [1-3]. *Flt-1$^{lacZ/+}$* mice (gift of Guo-Hua Fong, University of Connecticut) were maintained through WT mating. Ethical standards for animal use were maintained according to the University of North Carolina, Chapel Hill, Institutional Animal Care and Use Committee.

Inducible Cre Excision and Retina Tissue Processing

Maximal Cre-mediated gene excision was achieved by administering 100 μg of tamoxifen (MP Biomedicals) in 10% pure ethanol and 90% sunflower seed oil (Sigma) via daily intraperitoneal (IP) injections from postnatal day 2 (P2) to 4. Postnatal day 6 eyes were perfusion fixed with 0.5% paraformaldehyde (PFA) in PBS, collected, and immersed in 2% PFA for 2 hours at room temperature. Following PBS rinse, the retinal layer was isolated from fixed eyes by micro-dissection and immersed in cold, pure ethanol for 30 mins. Retinal tissues were re-hydrated and permeabilized for 30 mins in room temperature PBS-T, that is, PBS with 1% Triton-X (Fisher).

In samples from both conditional *flt-1* deletion mice and *flt-1*$^{lacZ/+}$ mice, blood vessels were labeled by incubating retinas in isolectinB4 conjugated to AlexaFluor 488 (1:100, Invitrogen). For beta-galactosidase immunostaining in *flt-1*$^{lacZ/+}$ samples, retinas were incubated with 5% normal donkey serum (Jackson ImmunoResearch) in PBS-T for 1 hour at room temperature, rabbit anti-beta-galactosidase (1:200, Invitrogen) in PBS-T overnight at 4°C, and donkey anti-rabbit AlexaFluor 633 (ThermoFisher) in PBS-T for 3 hours at room temperature. For pericyte/smooth muscle cell labeling, *flt-1*$^{lacZ/+}$ retinas were additionally incubated with Cy3-conjugated mouse anti-alpha smooth muscle actin (1:500, Sigma, clone 1A4). Following PBS washes, retinas were mounted in PBS:glycerol (1:1) and imaged using a Leica DMI 6000B or Zeiss LSM 880 confocal microscope at 40× or 63× magnification, with post-acquisition *z*-stacks compression using ImageJ software.

Quantitative Analysis of Retinal Vasculature

Tg(UBC$^{Cre\text{-}ERT2}$*)* retinas with maximal Cre-mediated excision of *flt-1* were analyzed for the percent overlap between TdTomato reporter expression (i.e. red fluorescence) and isolectinB4 labeling of blood vessels. In ImageJ, threshold cutoffs were applied to confocal images of both the isolectinB4 and TdTomato signals to reduce background noise and create binary images. The total area of isolectinB4+ vessels within a given region-of-interest was found, and then the “multiply” function was applied to both images to yield an image of pixels positive for both signals, thus facilitating measurement of isolectinB4+; TdTomato+ area. Percent overlap was then calculated by dividing isolectinB4+; TdTomato+ area by total isolectinB4+ area.

Embryonic Tissue Processing and Quantitative Analysis of Dermal Vasculature

Timed matings were established between *Cdh5-Cre; R26R*$^{TdTom/TdTom}$*; Flt-1*$^{loxP/+}$ males and *R26R*$^{TdTom/TdTom}$*; Flt-1*$^{loxP/+}$ female mice, and pregnant females were euthanized at embryonic day 12.5 (E12.5). Embryos were extracted from the uterine horns and immersion fixed in 4% PFA overnight at 4°C. After several rinses in PBS, full-thickness dermal tissue was dissected from the head and back regions of embryo proper. Tissues were immunostained for rat anti-mouse PECAM-1 (1:200, BD Biosciences) in PBS-T overnight at 4°C, and donkey anti-rat AlexaFluor 488 (ThermoFisher) in PBS-T for 3 hours at room temperature. Following DAPI staining for cell nuclei (1:1000 in PBS-T, 30 mins at RT) and PBS washes, skin samples were mounted in PBS:glycerol (1:1) and imaged using a Leica DMI 6000B confocal microscope at 20× or 40× magnification, with post-acquisition *z*-stacks compression using ImageJ software. Applying previously established methods [2,4], the sprouting vascular front of the E12.5 skin vasculature was analyzed for the density of endothelial filopodial extensions (i.e. per vessel length of the vascular front).

Cell Culture and Live Imaging

Maintenance and differentiation of *Flt-1*$^{-/-}$ (gift of Guo-Hua Fong, University of Connecticut) and WT ES cell was reported previously [5], as was the creation of *PECAM-1*^{eGFP} expressing ES cell lines [6]. Day 7-8 differentiating ES cell cultures were live imaged as follows: confocal images (20× objective) were acquired at 10 min intervals for 20 hrs with an Olympus FluoView FV1000 system, both with full environmental chamber. A z-stack of 6 images was acquired for each scan with 4 microns between focal planes, and was compressed into a

single image for each time step. Representative movie sequences shown are from non-consecutive images.

ES Cell-derived Vessel Antibody Staining

Day 8 WT- and *flt-1*$^{-/-}$-*PECAM-1*^{eGFP} ES cell cultures were fixed and stained as described [7]. AlexaFluor568-conjugated phalloidin was applied at 1:1000 to detect filamentous actin. Confocal images (40× objective) were acquired with a Leica DMI 6000B. A z-stack of 4-6 images was acquired for each scan with 0.7 μm between focal planes (1 Airy Unit). After acquisition, z-stacks were compressed into a single image for each genotype.

FIGURE LEGENDS

Supplementary Figure S1. TdTomato reporter expression indicates Cre-recombinase-mediated gene excision activity. (a-c) Representative images of postnatal day 6 (P6) *Tg(UBC$^{Cre\text{-}ERT2}$)* conditional *flt-1* knockout (KO) mouse retinas labeled for blood vessels (isolectinB4-AlexaFluor488 in a, and green in merged image c) and for the TdTomato excision reporter (b, and red in merged image c). Scale bar, 100 µm. **(d)** Average percentage of overlap of IsolectinB4+ signal with TdTomato+ signal within regions-of-interest from littermate control and conditional *flt-1* loss mice. Values are averages + standard deviation.

Supplementary Figure S2. Endothelial cell (EC)-specific *flt-1* deletion in embryonic skin disrupts endothelial cell sprouting. (a) Representative images of embryonic day 12.5 (E12.5) WT (top 2 rows) and *Cdh5-Cre flt-1* knockout (KO) (bottom 2 rows) mouse skin labeled for blood vessels (PECAM-1-AlexaFluor488 in the first column, and green in merged images of the third column) and for the TdTomato excision reporter (middle column, and red in merged images). Scale bars, 20 µm. **(b)** Graphs display counts of filopodia per 100 microns of vessel length at the vascular front of embryonic skin networks. Values are sums across multiple samples (n=4-6).

Supplementary Figure S3. Minimal to no *flt-1* expression detected in vascular mural cells of postnatal mouse retina vessels. (a-f) Representative images of postnatal day 8 (P8) *flt-1$^{LacZ/+}$* retinal vasculature labeled for blood vessels (isolectinB4-AlexaFluor488 in a, and green in merged images d and f), vascular mural cells [Cy3-alpha smooth muscle actin

(aSMA) in b, and red in merged images e and f], and *flt-1* promoter activity i.e. beta-galactosidase (beta-gal) production [beta-gal-Alexa Fluor633 secondary in c, and blue in merged images d, e and f]. Scale bar, 30 μm. White arrows indicate alpha-SMA+ mural cells along isolectinB4+ endothelial cells that contain no signal for beta-gal i.e. *flt-1* promoter activity.

Supplementary Figure S4. Endothelial cell sprouting dynamics in *WT; PECAM-1^{eGFP}* and *flt-1$^{-/-}$; PECAM-1^{eGFP}* embryonic stem (ES) cell-derived vessels. (a-f) Representative images of endothelial cell sprouts in *WT; PECAM-1^{eGFP}* (a-c) and *flt-1$^{-/-}$; PECAM-1^{eGFP}* (d-f) embryonic stem (ES) cell-derived vessels. Enhanced GFP (eGFP) expression by the *pecam-1* promoter facilitates visualization of endothelial cells (a and d, and green in the merged images c and f), and AlexaFluor568-conjugated phalloidin labels filamentous actin throughout the cultures, including the endothelial cells (b and e, and red in the merged images c and f). White arrowheads indicate the narrow cell extension and actin distribution in the WT sprout (a-c) as a comparison to the more widely distributed actin in the bifurcated *flt-1$^{-/-}$* sprout (d-f). **(g-k)** Representative sequential images from a movie of a sprouting and bifurcating *flt-1$^{-/-}$; PECAM-1^{eGFP}* ES cell-derived sprout. Black arrows point to the sprout initiation site (g) and the bifurcation of the sprout (i and j). Time (hrs:min), upper right. Scale bar, 20 μm.

Supplementary Figure S5. Nuclear Labelling (DAPI) of Sprout Endothelial Cells. From the images of retinal sprouts shown in Figure 4, non-endothelial and non-sprout nuclei (via DAPI stain) were masked out to define the number of cells involved in each sprout.

Isolated nuclei are shown in white (a-f) and overlaid with the blood vessel label (isolectinB4, green in a, c, d, and f) and with the excision reporter signal (TdTomato, red in b, c, e, and f) for each sprout configuration. Yellow arrows point to the nucleus associated with the tip cell for each sprout, and the yellow arrowheads indicate the nuclei of base cells within the sprouting region. Scale bar, 50 μm.

Supplementary Video S1. Live imaging of a bifurcating endothelial sprout in *flt-1$^{-/-}$; PECAM-1^{eGFP}* embryonic stem (ES) cell-derived vessels. From *Online Resource 4* of the Supplemental Materials, a time sequence of a *flt-1$^{-/-}$* mutant (*PECAM-eGFP*) sprout initiation from a parent vessel and subsequent bifurcation. Black arrows point to the sprout initiation site (g) and the bifurcation of the sprout (i and j). Time (hrs:min), upper left. Scale bar, 30 μm.

REFERENCES

1. Nesmith, J.E.; Chappell, J.C.; Cluceru, J.G.; Bautch, V.L. Blood vessel anastomosis is spatially regulated by Flt1 during angiogenesis. *Development* **2017**, *144*, 889-896, doi:10.1242/dev.145672.

2. Chappell, J.C.; Taylor, S.M.; Ferrara, N.; Bautch, V.L. Local guidance of emerging vessel sprouts requires soluble Flt-1. *Dev Cell* **2009**, *17*, 377-386, doi:S1534-5807(09)00295-0 [pii] 10.1016/j.devcel.2009.07.011.

3. Ambati, B.K.; Nozaki, M.; Singh, N.; Takeda, A.; Jani, P.D.; Suthar, T.; Albuquerque, R.J.; Richter, E.; Sakurai, E.; Newcomb, M.T., et al. Corneal avascularity is due to soluble VEGF receptor-1. *Nature* **2006**, *443*, 993-997.

4. Hellstrom, M.; Phng, L.K.; Hofmann, J.J.; Wallgard, E.; Coultas, L.; Lindblom, P.; Alva, J.; Nilsson, A.K.; Karlsson, L.; Gaiano, N., et al. Dll4 signalling through Notch1 regulates formation of tip cells during angiogenesis. *Nature* **2007**, *445*, 776-780, doi:nature05571 [pii] 10.1038/nature05571.

5. Kearney, J.B.; Bautch, V.L. In vitro differentiation of mouse ES cells: hematopoietic and vascular development. *Methods Enzymol* **2003**, *365*, 83-98.

6. Kearney, J.B.; Kappas, N.C.; Ellerstrom, C.; DiPaola, F.W.; Bautch, V.L. The VEGF receptor flt-1 (VEGFR-1) is a positive modulator of vascular sprout formation and branching morphogenesis. *Blood* **2004**, *103*, 4527-4535.

7. Kappas, N.C.; Zeng, G.; Chappell, J.C.; Kearney, J.B.; Hazarika, S.; Kallianos, K.G.; Patterson, C.; Annex, B.H.; Bautch, V.L. The VEGF receptor Flt-1 spatially modulates Flk-1 signaling and blood vessel branching. *J Cell Biol* **2008**, *181*, 847-858, doi:jcb.200709114 [pii] 10.1083/jcb.200709114.
